# Novel HTS Strategy Identifies TRAIL-Sensitizing Compounds Acting Specifically Through the Caspase-8 Apoptotic Axis

**DOI:** 10.1371/journal.pone.0013375

**Published:** 2010-10-12

**Authors:** Darren Finlay, Robyn D. Richardson, Lisa K. Landberg, Amy L. Howes, Kristiina Vuori

**Affiliations:** Cancer Center, Sanford-Burnham Medical Research Institute, La Jolla, California, United States of America; University of Illinois at Chicago, United States of America

## Abstract

Tumor Necrosis Factor-Related Apoptosis-Inducing Ligand (TRAIL) is potentially a very important therapeutic as it shows selectivity for inducing apoptosis in cancer cells whilst normal cells are refractory. TRAIL binding to its cognate receptors, Death Receptors-4 and -5, leads to recruitment of caspase-8 and classical activation of downstream effector caspases, leading to apoptosis. As with many drugs however, TRAIL's usefulness is limited by resistance, either innate or acquired. We describe here the development of a novel 384-well high-throughput screening (HTS) strategy for identifying potential TRAIL-sensitizing agents that act solely in a caspase-8 dependent manner. By utilizing a TRAIL resistant cell line lacking caspase-8 (NB7) compared to the same cells reconstituted with the wild-type protein, or with a catalytically inactive point mutant of caspase-8, we are able to identify compounds that act specifically through the caspase-8 axis, rather than through general toxicity. In addition, false positive hits can easily be “weeded out” in this assay due to their activity in cells lacking caspase-8-inducible activity. Screening of the library of pharmacologically active compounds (LOPAC) was performed as both proof-of-concept and to discover potential unknown TRAIL sensitizers whose mechanism is caspase-8 mediated. We identified known TRAIL sensitizers from the library and identified new compounds that appear to sensitize specifically through caspase-8. In sum, we demonstrate proof-of-concept and discovery of novel compounds with a screening strategy optimized for the detection of caspase-8 pathway-specific TRAIL sensitizers. This screen was performed in the 384-well format, but could easily be further miniaturized, allows easy identification of artifactual false positives, and is highly scalable to accommodate diverse libraries.

## Introduction

Tumor Necrosis Factor-Related Apoptosis-Inducing Ligand (TRAIL) is of great potential use as an anti-cancer therapeutic, however, its evaluation and use as a potential therapeutic agent have been limited by resistance. TRAIL selectively induces apoptosis in cancer cells whilst normal cells are refractory [1]. It is safe when administered *in vivo* and shows none of the toxicity usually associated with other members of the Tumor Necrosis Factor (TNF)-superfamily [2]. TRAIL is a naturally occurring cytokine that canonically acts by binding as a homotrimer to Death Receptor (DR)-4 or -5, resulting in recruitment of adapter molecules such as FADD and caspase-8. This recruitment and clustering results in caspase-8 dimerization, activation, processing and release from the complex. Activated caspase-8, in turn, activates the effector caspases resulting in classical apoptotic cell death [3]. DR4 and DR5 are widely expressed on cancer cells and it has been suggested that this may be one reason for TRAIL's anti-tumor properties [4]. However, a full understanding of the mechanisms of TRAIL selectivity remains elusive. TRAIL has also been demonstrated to bind to two “decoy receptors”, DcR1 and DcR2. Both of these receptors lack intracellular domains capable of transducing apoptosis and may also account for the selectivity and/or resistance associated with TRAIL signaling.

As TRAIL resistance is a major preclusion to its clinical use, we envisaged a novel high-throughput screen (HTS) to identify sensitizing agents. As non-specificity and toxicity are a major drawback with conventional chemotherapeutics, we proposed to discover novel agents acting primarily through the caspase-8 axis as opposed to generalized toxicity. Utilization of the caspase-8 null neuroblastoma cell line, NB7, and the same line reconstituted with either wild-type or a catalytically inactive point mutant of caspase-8 (C360A) [5] in parallel allows the identification of chemical agents that act specifically via the caspase-8 apoptotic pathway. The use of all three cell lines in parallel will allow easy identification of false positives that induce non-specific cell death.

Concomitantly, we investigated the ability of chemical compounds to sensitize or potentiate caspase-8-induced cell death in response to low concentrations of TRAIL. For proof-of-concept analysis, the library of pharmacologically active compounds (LOPAC) of 1280 compounds was chosen due to its relatively small size and the fact that all agents have biological activity. We propose that this pilot screen, using in parallel the three cell lines discussed above, provides us with proof-of-concept for a larger screening campaign to follow. Indeed, while the use of cells lacking functional caspase-8 may seem redundant, they are included in the primary screen here due to the “proof-of-concept” nature of the initial screen and should allow for more rigorous identification of any anomalies. Larger and more relevant chemical libraries will be screened only against cells with functional caspase-8 whilst the cells lacking the enzyme will be utilized in the secondary confirmations.

We demonstrate development of a novel 384-well HTS that allows simple identification of generally toxic “false positives”. Further, we have detected known TRAIL synergizers and previously undescribed sensitizing agents. The system is semi-automated and highly scalable so that vastly greater compound numbers can easily be tested.

## Results

**Figure 1:**
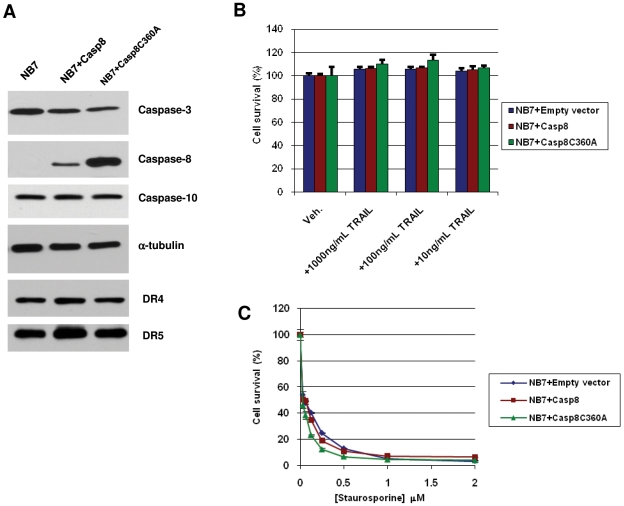
NB7 cells are TRAIL resistant yet possess intact apoptotic machinery. A) Immunoblot analysis of apoptotic protein expression in NB7 cells. B) Cell survival assay (MTS) on NB7 cells treated with TRAIL as described (24 h). C) Cell survival assay (MTS) on NB7 cells treated with staurosporine (24 h). All dose response curves provided were carried out in at least triplicate at least three independent times and a representative graph is shown. Data values are averages +/− S.E.M. We note the S.E.M. values for some samples are extremely small and therefore may be difficult to see in the graph.

Many cancer cells are resistant to TRAIL, yet the exact mechanisms are unknown and probably vary widely (e.g. [6]). Epigenetic silencing of caspase-8 has been reported for several different cancer cells and caspase-8 re-expression can restore TRAIL-mediated killing [7], [8]. We show that the neuroblastoma NB7 cells are deficient in caspase-8 (Fig. 1A, [9], [10]) yet express death receptors DR4 and DR5 and caspases -3 (an effector caspase) and -10 (an apical caspase). Interestingly, these cells are refractory to TRAIL-induced apoptosis even when caspase-8 is re-expressed (Fig. 1B). The cells are, however, sensitive to apoptosis induced by the serine-threonine kinase inhibitor staurosporine, indicating that the underlying apoptotic effector machinery is intact (Fig. 1C). We further note that staurosporine kills NB7 cells in a caspase-8 independent manner. Thus, our cells are a suitable model system for the identification of novel TRAIL-sensitizing agents acting through the caspase-8 axis.

**Figure 2:**
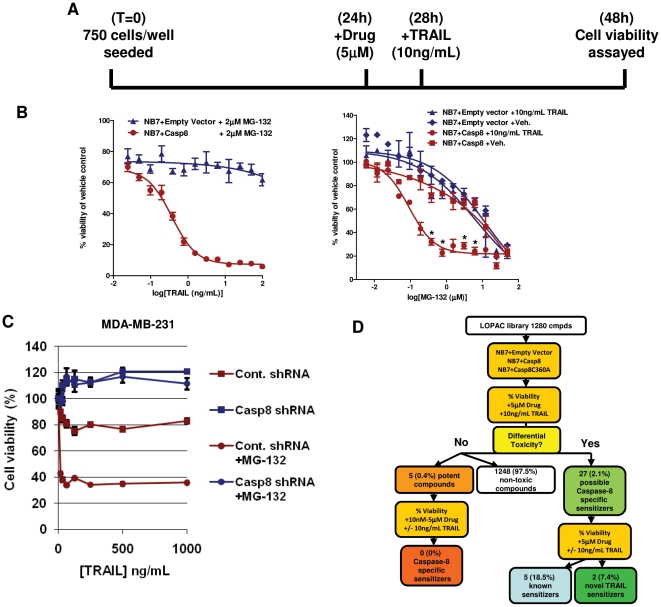
MG-132 acts as TRAIL sensitizer in NB7 cells with caspase-8 re-constituted. A) Schematic of experimental time-line. B) Cell survival assays (CellTiter-Glo) on NB7 and NB7+Casp8 cells pre-treated for 4 h with 2 µM MG-132 before treatment with TRAIL as described for 20 h (left panel) or the same cells treated with varying concentrations of MG-132 before addition of TRAIL (10 ng/mL) as above. Data values are averages +/− S.E.M. C) Cell survival assay of MDA-MB-231+Control shRNA and MDA-MD-231+Casp8 shRNA cells pretreated with either vehicle or MG-132 (2 µM) before addition of TRAIL for 20 h. Data values are averages +/− S.E.M. We note the S.E.M. values for some samples are extremely small and therefore may be difficult to see in the graph. D) Work flow of hit rates, confirmations and retesting of compounds.

As proteosome inhibition has proven to be effective in the treatment of certain cancers, we first investigated whether MG-132 would sensitize NB7 cells re-expressing caspase-8 (NB7+Casp8 cells) to TRAIL-induced apoptosis. Pre-treatment of the cells with 2 µM MG-132 for 4 hours prior to 20 hours of treatment with TRAIL (e.g. Fig. 2A) results in apoptosis of approximately 90% of the NB7+Casp8 cells (even with only 3.125 ng/mL TRAIL) while the cells deficient in caspase-8 remain refractory (Fig. 2B). We further show that the same assay can be applied to breast cancer cells expressing a silencing caspase-8 shRNA or a non-silencing scrambled control. The majority of the cells are refractory to TRAIL even at high concentrations yet MG-132 again acts as a sensitizing agent in a caspase-8 dependent manner (Fig. 2C). The MDA-MB-231 breast cancer cells were chosen as they have been shown to express TRAIL receptors and to possess intact downstream apoptotic machinery [11]. Furthermore, these cells allow us to demonstrate functionality in another cell type and to utilize shRNA as another method to “knock down” the protein of interest. Thus our system and protocols should be readily transferable to investigate other systems and targets. The ease with which the assay could be successfully miniaturized to 384-well format suggests automation and further miniaturization to a 1536-well format should be possible, while maintaining sensitivity and statistical rigor. This is extremely important as the costs associated with screening large chemical libraries are not inconsiderate.

For the screen itself, a luminescent read-out for cell viability (CellTiter-Glo® Luminescent Cell Viability Assay, Promega Corp., Madison, WI) was chosen as the primary measure because there is generally less spectral interference by test compounds using luminescence than with absorbance-based viability assays. Z' factors take into account the variability and dynamic range of the assay signal across entire plates and are used to judge the statistical significance of each individual well. In general, cellular assays with Z'≥0.4 are considered sufficiently rigorous for single measurements. Our assay greatly exceeds this benchmark with Z' = 0.73 – 0.85 for the three tested cell lines. Screened compounds were assessed for their ability to kill NB7+Casp8 cells more potently in the presence of TRAIL. MG-132 was utilized as a positive control in our system. The LOPAC library was screened at a concentration of 5 µM against the three cell lines in the presence of 10 ng/mL TRAIL. Compounds were considered potential TRAIL sensitizers if viability in the NB7+Casp8 + TRAIL condition was less than 50% of the vehicle-only control and at least 20% less than the viability of the NB7 + TRAIL and/or NB7+Casp8C360A + TRAIL conditions. “Potent” compounds were defined as those compounds that resulted in cell viability of less than 15% as compared to the vehicle-only control under all test conditions (wherein sensitization is masked due to high toxicity at 5 µM). The library contained altogether 32 (2.5%) potential caspase-8-dependent, TRAIL sensitizing agents, of which five were termed as “potent” compounds due to observed killing of all three cell lines, based on the criteria described above.

Of the potent compounds, four (Bay 11-7085, 4-aminopyridine, DL-erythro-dihydrosphingosine, and β-lapachone) were retested over a lower concentration range in the presence or absence of TRAIL but none were confirmed as sensitizing agents in our system. The fifth potent compound, DL-alpha-methyl-p-tyrosine, was unavailable for purchase and was dropped from the campaign.

A secondary screen of the remaining 27 potential caspase-8 specific hits was performed by testing the compounds at 5 µM in six conditions consisting of all three cell lines in the absence or presence of TRAIL. This secondary screen served two purposes: a) to confirm the primary screen results and b) to assay the extent, if any, of TRAIL sensitization in the context of caspase-8 or the catalytically inactive point mutant, where an attenuated, but measurable, effect is seen with MG-132. This retesting confirmed that seven compounds were true caspase-8 axis TRAIL sensitizers. A comprehensive search of the literature revealed that five of the compounds (camptothecin, genistein, paclitaxel, wortmannin, and thapsigargin) had been previously reported as TRAIL synergizers [12]–[16] and two (YC-1 and APDC) are previously unidentified TRAIL-sensitizing compounds (Table 1). Compounds dropped from the campaign at this point consisted of those that did not reconfirm (11 compounds) and those that lacked significant sensitization to TRAIL (cell viability in the presence of the compound + TRAIL is greater than 80% of compound only-treated cells; nine compounds). A work flow of hit rates, confirmations and retesting of the compounds is shown in Fig. 2D.

**Table 1:** 

Compound	Reported Mechanism	Viability*
Camptothecin	Topo-I inhibitor	37.1%
Genistein	Tyrosine kinase inhibitor	14.9%
Paclitaxel	Microtubule disassembly inhibitor	43.9%
Wortmannin	PI3-K inhibitor	39.0%
Thapsigargin	SERCA inhibitor	20.1%
APDC	mGluR II agonist	29.2%
YC-1	Guanylate cyclase activator	30.6%

*Viability is defined as % cell survival of NB7+Casp8 cells treated with drug (5 µM) +20 ng/mL TRAIL as compared to cells treated with vehicle (data from original screen).

**Figure 3:**
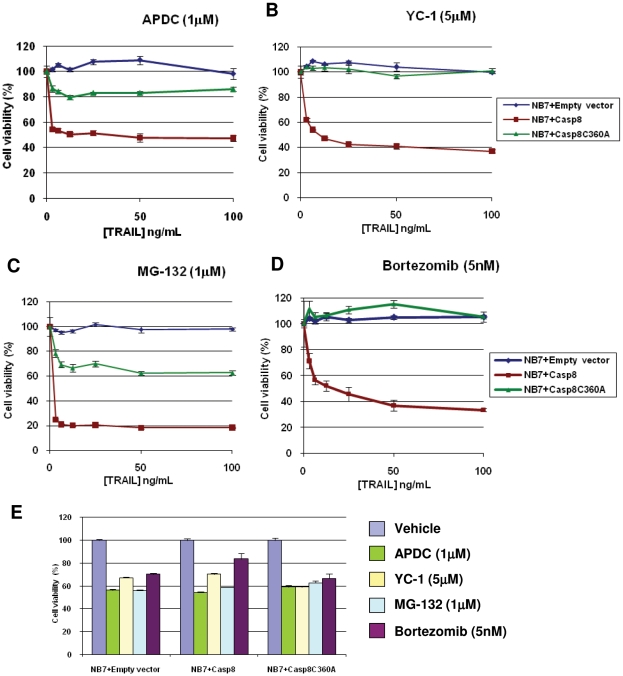
Cell survival TRAIL dose response curves. Cell viability measurements (CellTiter-Glo) on NB7 cells treated with A) 1 µM APDC (Ammonium pyrrolidinedithiocarbamate), B) 5 µM YC-1 [3-(5′-Hydroxymethyl-2′-furyl)-1-benzyl indazole], C) 1 µM MG-132 or D) 5 nM Bortezomib for 4 h and TRAIL for 20 h as described. Cell viability is normalized to that of compound alone (APDC, YC-1, MG-132 and Bortezomib, respectively). E) Single agent toxicity (CellTiter-Glo) on NB7 cells treated with drug alone for 24 h. All dose-response curves were carried out in at least triplicate at least three independent times. A representative graph is shown. Data values are averages +/− S.E.M.

Activity of YC-1 and APDC was re-analyzed by TRAIL dose-response (Fig. 3A–D). As described previously, NB7 cells and those reconstituted with the wild-type, or a catalytically inactive mutant of caspase-8 were pretreated for 4 hours with APDC (1 µM), YC-1 (5 µM), MG-132 (1 µM) or the clinically-relevant proteosome inhibitor Bortezomib (5 nM). Cells were then incubated with TRAIL for 20 hours before cell viability was assessed via the CellTiter-Glo assay. APDC, YC-1 and MG-132 were confirmed as novel TRAIL sensitizing agents whilst the FDA-approved agent, Bortezomib, demonstrated caspase-8-dependent TRAIL-sensitization at 5 nM. Although the native CASP10 gene is intact and caspase-10 protein is expressed in the NB7 cells (Fig. 1A), it does not appear to be involved in this modulation of TRAIL-induced apoptosis.

**Figure 4:**
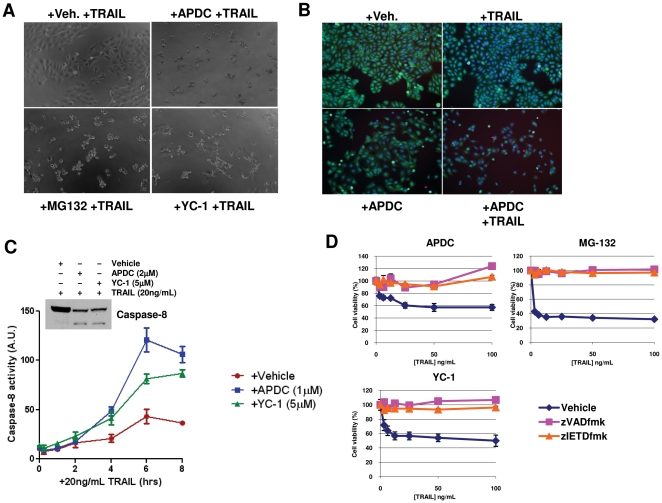
TRAIL-sensitized cell death results in the morphological signs of apoptosis. A) Phase contrast images (10x magnification) of NB7+Casp8 cells treated with vehicle, 1 µM APDC, 1 µM MG-132 or 5 µM YC-1 before addition of 20 ng/mL TRAIL for 20 h. B) LIVE/DEAD ® Viability/Cytotoxicity assay of cells treated with either vehicle, 1 µM APDC, 20 ng/mL TRAIL or both the APDC and TRAIL combined, whereby green fluorescence signifies live and red fluorescence signifies dying cells. C) Caspase-Glo 8 assay time course of cells treated with vehicle, 1 µM APDC or 5 µM YC-1 before addition of 20 ng/mL TRAIL for the indicated time. Data values are averages +/− S.E.M. Inset in C) Immunoblot of caspase-8 processing in NB7+Casp8 cells treated with vehicle, APDC (2 µM) or YC-1 (5 µM) for 4 h before addition of TRAIL (20 ng/mL) for 18 h. D) Cell viability measurements (CellTiter-Glo) on NB7+Casp8 cells treated with 1 µM APDC, 5 µM YC-1, or 1 µM MG-132 in the presence and absence of the small peptide caspase inhibitors, zIETD-fmk or zVAD-fmk (both 50 µM), for 4 h and TRAIL (20 ng/mL) for 20 h.

Phase contrast images of NB7+Casp8 cells treated with vehicle, APDC, MG-132 or YC-1 before addition of TRAIL for 20 h as before are shown in Fig. 4A. Cells treated with vehicle and TRAIL as described appeared healthy while cells pretreated with the other three agents followed by TRAIL treatment demonstrated a condensed phenotype typical of a dying cell. These cells do not appear to die via anoikis, as only little detachment takes place (Fig. 4A), however the small number of detached cells are dead as assessed by ethidium homodimer uptake. In addition to the loss of cell viability measured by CellTiter-Glo, cell death can also be detected in NB7+Casp8 cells after treatment with APDC followed by TRAIL as described previously. APDC sensitization to TRAIL-induced killing was assessed using LIVE/DEAD ® Viability/Cytotoxicity Kit (Molecular Probes) whereby green fluorescence signifies live cells (Fig. 4B). We note that cells treated with vehicle or TRAIL alone are healthy and cells treated with APDC alone show only minor cell loss while maintaining the green fluorescence associated with live cells. In turn, cells treated with APDC followed by TRAIL results in a marked loss of cell number coupled with reduced green “live cell” signal intensity. A few red fluorescing (dying) cells are also observed, however it is of note that dead, unattached cells will fail to produce red fluorescence, resulting in less red signal observed.

To further investigate the putative role of the agents in promoting caspase-8 activity, we assessed the ability of the agents to sensitize the NB7+Casp8 cells to TRAIL-mediated LETD peptide cleavage using the Caspase-Glo 8 assay system (Promega Corp.). Cells were treated with APDC (1 µM), YC-1 (5 µM) or vehicle control as described before addition of 20 ng/mL TRAIL and a time course of caspase-8 activation is presented in Fig. 4C. We also show by immunoblot analysis that TRAIL treatment results in caspase-8 processing only when the cells are pre-treated with APDC or YC-1, but not with vehicle alone (Fig. 4C). As such, both agents demonstrate the ability to promote TRAIL-mediated caspase-8 activation, suggesting that they likely act on upstream molecules of the pathway. We also demonstrate that the small peptide inhibitors of caspase-8 (zIETD-fmk) and the pan-caspase inhibitor, zVAD-fmk, provide complete protection from the TRAIL killing sensitized by APDC (1 µM), YC-1 (5 µM) and MG-132 (1 µM) (Fig. 4D).

In sum, we describe here a cellular model of TRAIL resistance that is retained even upon caspase-8 re-expression. We also show the design and optimization of a 384-well high throughput assay that is highly scalable. By screening the LOPAC library using this assay, we identified five known and two previously undescribed TRAIL-sensitizing agents that are confirmed with concentration-dependent curves produced from cell viability measurements.

## Discussion

Current cancer chemotherapeutics are significantly limited by a lack of selectivity for the intended target and deleterious side effects. While targeted agents such as kinase inhibitors or antibodies offer greatly more selectivity, they are susceptible to inherent or acquired resistance. Therefore targeted apoptosis is an attractive methodology for removal of the supernumerary cells associated with cancer progression. One potentially valuable agent is TRAIL. As mentioned previously, TRAIL shows selectivity for cancer cells, but resistance remains a problem. Resistance due to up-regulation of the decoy receptors can be simply overcome by using targeted agonistic antibodies. Indeed, antibodies that target specific death receptors and have little-to-no affinity for the decoy receptors may prove to be more potent and selective than TRAIL itself. Even with highly specific agonistic antibody therapy however, resistance may still occur due to intracellular alterations in the apoptotic cascade. In keeping with a small molecule pharmacological approach, we describe here a novel screening strategy optimized for the detection of caspase-8 pathway-specific TRAIL sensitizers, and demonstrate its usefulness by a proof-of-concept screen.

Caspase-8 is an apical protease involved in the “extrinsic” or death receptor-mediated form of cell death and, as such, would seem to be an ideal candidate for silencing or deletion in cancers. Surprisingly, however, the protein is rarely silenced or absent in cancers, making it an extremely attractive therapeutic intervention point [10]. By utilizing a cell system that lacks caspase-8 (NB7) compared to cells with the functional protein re-expressed, we are able to differentiate between sensitizing agents that act through the caspase-8 pathway as opposed to agents acting through other mechanisms. We show that while NB7 cells express DR4 and DR5, they remain TRAIL resistant, even when functional caspase-8 is re-expressed (Fig. 1B). This is likely due to alterations in the intracellular apoptotic or survival pathway machinery. Indeed, the FDA-approved proteosome inhibitor Bortezomib has been shown to act in part by preventing IκB degradation, thereby blocking NF-κB mediated cell survival. Similarly, we demonstrate that proteasome inhibition with MG-132 or Bortezomib results in TRAIL sensitization in a caspase-8 specific manner (Fig. 2 and 3). Although NB7+Casp8 cells are resistant to TRAIL concentrations up to 1 µg/mL (Fig. 1B), a 4 hour pre-treatment with 2 µM MG-132 allows 90–95% killing with less than 10 ng/mL TRAIL (Fig. 2). We note, however, that caspase-8 re-expression does not result in differential sensitivity to the other non-specific apoptosis inducers such as the serine threonine kinase inhibitor staurosporine (Fig. 1C). Thus, our model system truly allows identification of TRAIL-sensitizing agents acting selectively via the caspase-8 apoptotic axis, as re-expression of the protein alone has no effect on generalized toxicity. The objective of our screen was to show that compounds that sensitize cells to TRAIL-mediated death, and whose mechanism involves caspase-8 in some way, could be identified by screening the three neuroblastoma lines in parallel and in the presence of TRAIL. To this end, we screened the 1280 compound LOPAC collection and indeed, all five known TRAIL sensitizers identified from the screen (camptothecin, genistein, paclitaxel, wortmannin, and thapsigargin) have known mechanisms of action involving caspase-8 [12]–[16].The entirety of the screen was performed in the presence of TRAIL for two reasons: 1) All three of the neuroblastoma lines used in the screen are completely refractory to the levels of TRAIL used in the screen, which is to say that the viability signal from “vehicle + TRAIL” wells did not significantly differ from that from “vehicle – TRAIL” and so could be used to define the high end of the dynamic range of signal without running additional conditions in parallel, which would add cost to the assay and 2) that, by definition, a “hit” in the screen should be more potent in the presence of TRAIL, thereby lowering the signal in the corresponding well on only the NB7+Casp8 plate, thus increasing the sensitivity of the assay. We are aware that the set-up of the primary screen utilized here does not differentiate between compounds that act through the caspase-8 axis and those that do so and also sensitize with TRAIL. In the current work, we address this question in the secondary assay where hits from the primary screen were tested in the presence and absence of TRAIL; however, depending on the size and content of the library, the condition of the NB7+Casp8 cells with PBS instead of TRAIL can easily be added to the screen, or run instead of the point mutant condition. In fact, we believe the tailorability of this assay is an asset because a screen can be performed to find compounds of specific action.

As TRAIL is a potentially important cancer therapeutic, many laboratories have attempted to find sensitizing agents utilizing a wide range of chemical libraries against various different cell types. Similar studies by other investigators utilizing a renal cell carcinoma model to search for TRAIL-sensitizing agents [17] have also demonstrated that novel agents of this class can be identified by such a strategy. These studies also confirmed MG-132 as a TRAIL-sensitizing agent. Other strategies have involved assessing the ability of known anti-cancer agents to act as TRAIL-sensitizing agents. Treatment of TRAIL resistant hepatocellular carcinoma cells with specific kinase inhibitors or known chemotherapeutic drugs revealed that both classes of agent could sensitize to TRAIL in a caspase-dependent manner [18]. Another study of the effects of known chemotherapeutic agents in two established ovarian cancer cell lines, however, showed that cisplatin and paclitaxel had no TRAIL-sensitizing abilities [19]. This investigation did however identify the flexible heteroarotinoid compound SHetA2 as an extrinsic apoptosis sensitizing agent. Thus the testing of potential TRAIL sensitizers against cells of various tissue types or against cancer cell lines harboring genetic deletions, mutations or amplifications of clinical significance may aid clinicians in assessing if certain agents may prove more effective in certain cancer types. The previously unidentified sensitizers from our screen (Table 1) include APDC (ammonium pyrrolidinedithiocarbamate) and YC-1 (3-(5′-hydroxymethyl-2′-furyl)-1-benzyl indazole). APDC was originally described as a selective group II mGluR agonist and YC-1 as a soluble guanylate cyclase activator. At present time, it remains unknown whether these roles are related to their TRAIL-sensitizing abilities. Interestingly, YC-1 has also been reported to block HIF1α activity [20] and therefore may also operate at a transcriptional level.

In sum, we demonstrate the development of a 384-well HTS that allows identification of TRAIL-sensitizing agents acting specifically through the caspase-8 apoptotic pathways. The assay is highly automated, cost effective, amenable to multi-plexing analysis such as fluorescence-based caspase activation, and allows simple identification of false positive hits. We identified five known sensitizing agents from the LOPAC library along with two previously undescribed sensitizing agents. Future work will involve screening of larger and more relevant chemical compound libraries with a view to identifying lead compounds of potential clinical significance. In addition, screening of the F.D.A.-approved oncology drugs may provide insights into the clinical use of known agents, such as Bortezomib, as potential TRAIL sensitizers.

## Materials and Methods

### Reagents

Unless otherwise specified, all reagents were from Sigma-Aldrich (St. Louis, MO). Primocin was obtained from InvivoGen (San Diego, CA), Bortezomib from LC Laboratories (Woburn, MA), and MG-132 and TRAIL are from Calbiochem (La Jolla, CA). Small peptide caspase inhibitors are from BD Biosciences (La Jolla, CA).

### Preparation of cellular protein extracts

Cells were treated as described for experimental conditions. Media supernatants were removed by vacuum and the cells washed in ice-cold PBS. The cells were scraped in 1 mL of ice-cold PBS and pelleted by centrifugation at 20,000 g for 2 min at 4°C. Pellets were then re-suspended in an appropriate volume of cell lysis buffer containing 40 mM Tris (pH 8.0), 200 mM NaCl, 0.2% Triton X-100, 10% glycerol, 2 mM β-glycerophosphate, 500 µM NaF and a protease inhibitor cocktail (#1 697 498, Roche Diagnostics, Mannheim, Germany) and incubated on ice for 10 min with occasional agitation. The suspension was clarified by centrifugation at 20,000 g for 10 min at 4°C to yield supernatants comprising the total cell extract. Protein concentrations were determined using a modified method of Bradford [21].

### Immunoblotting analysis

Protein extracts were resolved on SDS-polyacrylamide gels and then electrophoretically transferred onto PVDF membranes (Immobilon-P, Millipore, Temecula, CA) by standard methodology. Membranes were blocked for 1 h in TBS-T (20 mM Tris-HCl, pH 7.6, 150 mM NaCl, 0.05% Tween) containing 5% (w/v) non-fat dried milk and were incubated rocking overnight at 4°C with the appropriate primary antibody: anti-α-tubulin (DM1A, 1∶4000) (Calbiochem, La Jolla, CA); anti-caspase-8 (C15, 1∶500) (kind gift from Dr. Marcus Peter, University of Chicago, Chicago, IL); anti-caspase-10 (ab2012, 1∶2000) (Novus Biologicals); anti-caspase-3 (#610322, 1∶2000) and anti-DR4 (#556544, 1∶1000) (both from BD Biosciences, La Jolla, CA) and anti-DR5 (#3696, 1∶1000) (Cell Signaling Technology, Danvers, MA). After incubation for 1 h with anti-rabbit IgG (111-035-003), or -mouse IgG (115-035-003) secondary antibodies conjugated to horseradish peroxidase (Jackson ImmunoResearch Laboratories Inc., West Grove, PA), bands were visualized using enhanced chemiluminescence (SuperSignal® West Pico Chemiluminescent substrate, #34080, Pierce, Rockford, IL).

### Cell culture and HTS design

Caspase-8 deficient NB7 cells were a kind gift from Dr. Jill Lahti (St. Jude Children's Research Hospital, Memphis, TN) and were maintained in RPMI 1640 supplemented with 10% (v/v) FBS, and penicillin/streptomycin/L-Glutamine (Omega Scientific Inc, Tarzana, CA). Stable cell lines expressing caspase-8 or a proteolytically inactive protein are as described previously [10], [22].

The initial screen of the LOPAC 1280 library (Sigma) was performed in 384-well white, clear-bottom TC plates (Greiner Bio-One, Monroe, NC) using luminescence as the primary read-out for cellular viability as measured with the CellTiter-Glo® Luminescent Cell Viability Assay (Promega Corp., Madison, WI). Neuroblastoma cells from the NB7, NB7+Casp8, or NB7+Casp8C360A lines were seeded at 750 cells/well in 50 µL complete medium and allowed to attach overnight. Test compounds were then added via an ECHO 550 acoustic pipettor (Labcyte, Sunnyvale, CA) at 25 nL/well for a final compound concentration of 5 µM and a final DMSO concentration of 0.03% (v/v). The plates were incubated for 4 hours, at which time TRAIL was added in PBS at 10 µL/well for a final concentration of 10 ng/mL via a MultiDrop Combi (Thermo Fisher Scientific, Waltham, MA). The plates were incubated an additional 20 hours, then CellTiterGlo reagent was added via MultiDrop Combi at 25 µL/well. The plates were orbitally shaken for 15 minutes and the resulting luminescence recorded with an Envision multimode plate reader (Perkin Elmer, Waltham, MA). Z'-factors were independently calculated for each cell line using luminescence from vehicle-treated cells as high signal and that from vehicle-treated medium as the low signal [23].

### Cell survival assays

MTS assays were carried out using Promega Corporation's (Madison, WI) CellTiter 96 AQueous Non-Radioactive Cell Proliferation Assay exactly as per manufacturer's instructions. Briefly, cells are treated as described before addition of 1/5 volume of assay reagent. Cells are then incubated at 37°C for 1 h before absorbance (490 nm) is read on a Biotek EL800 plate reader (Biotek, Winooski, VT).

### Hoechst and LIVE/DEAD staining

LIVE/DEAD® Viability/Cytotoxicity Kit (Molecular Probes, Eugene, OR) was used as per manufacturer's instructions. Cells were plated in 6-well dishes, and treated 24 hours later with 5 µM MG-132 or APDC, as indicated in figure. Subsequently, four hours later, 20 ng/ml TRAIL was added as appropriate. Following a further 20 hour incubation, the cells were incubated with live/dead stains (L-3224, Invitrogen, Carlsbad, CA). Specifically, the cells were incubated with 1 µM Calcein-AM, 2 µM ethidium homodimer, and 100 nM Hoechst 33342 for 30 minutes at 37°C. Fluorescence was then imaged using confocal microscopy.

### Caspase-Glo 8 assay

The Caspase-Glo 8 Assay (Promega Corp., Madison, WI) system was used as per manufacturer's instructions. Briefly, cells are treated as described before addition of 1/2 volume of assay reagent. Lysates are then incubated at room temperature for 1 h before luminescence is measured on a Biotek Synergy 2 plate reader.
